# Relationship between muscarinic M_1_ receptor binding and cognition in medication-free subjects with psychosis^☆^

**DOI:** 10.1016/j.nicl.2018.02.030

**Published:** 2018-03-03

**Authors:** Geor Bakker, Claudia Vingerhoets, Daphne Boucherie, Matthan Caan, Oswald Bloemen, Jos Eersels, Jan Booij, Thérèse van Amelsvoort

**Affiliations:** a Department of Psychiatry & Psychology, University of Maastricht, The Netherlands; b Department of Radiology and Nuclear Medicine, Academic Medical Center, University of Amsterdam, Amsterdam, The Netherlands; c GGZ Centraal, Center for Mental Health Care Innova, Amersfoort, The Netherlands

## Abstract

**Background:**

It is still unclear which underlying mechanisms are involved in cognitive deficits of psychotic disorders. Pro-cognitive effects of muscarinic M_1_ receptor agonists suggest alterations in M_1_ receptor functioning may modulate these symptoms. Post mortem studies in patients with schizophrenia have shown significantly reduced M_1_ receptor expression rates in the dorsolateral prefrontal cortex (DLPFC) compared to controls. To date no in-vivo examinations of M_1_ receptor binding in relation to cognitive impairments have been done. As cognitive deficits have similar course and prognostic relevance across psychotic disorders, the current study assessed M_1_ receptor binding in the DLPFC and hippocampus in relation to cognitive functioning.

**Methods:**

Muscarinic M_1_ receptor binding potential (BP_ND_) was measured using ^123^I-IDEX, single photon emission computed tomography (SPECT) in 30 medication-free subjects diagnosed with a psychotic disorder. A computerized neuropsychological test battery was used to assess cognition, and the positive and negative syndrome scale (PANSS) to assess severity of psychotic symptoms.

**Results:**

Assessment of cognitive domains showed that lower M_1_ BP_ND_ in the DLPFC was related to overall lower performance in verbal learning and memory. In addition, lower M_1_ BP_ND_ in the DLPFC was related to greater negative symptom severity. Lastly, lower M_1_ BP_ND_ in the hippocampus was related to worse delayed recognition of verbal memory.

**Conclusion:**

This is the first study to show that variation in M_1_ receptors in the DLPFC is related to cognitive and negative symptom outcome in psychotic disorders. The M_1_ receptor may be an important biomarker in biological stratification of patients with psychotic disorders.

## Highlights

• Lower DLPFC M_1_ BP_ND_ was related to lower overall performance in verbal learning and memory and negative symptoms severity
• Lower M_1_ BP_ND_ in the hippocampus was related to worse delayed recognition of verbal memory
• Results support the M_1_ receptor as important therapeutic target in treatment of psychotic symptoms

## 1 Introduction

An estimated 80% of subjects with psychotic disorders suffer from cognitive deficits, scoring 1–2 standard deviations below their peers (Green et al., 2004; Woodberry et al., 2008). These symptoms are already present in the prodromal phase and persist even after other symptoms have remitted (Green et al., 2004). Multiple cognitive domains are affected with most prominent deficits being reported in processing speed, attention and vigilance, working memory, verbal and visual learning and memory, reasoning and problem solving (Rodríguez-Jiménez et al., 2012). Severity of cognitive deficits best predict poor functional outcome and relapse, although this finding is supported primarily by studies done in schizophrenia (Kahn and Keefe, 2013). Currently, these symptoms cannot be treated adequately with available antipsychotics giving an urgent need to understand their underlying neuropathology (Vingerhoets et al., 2013).

Aggravation of cognitive impairments in patients with psychotic disorders by anti-muscarinic agents given to reduce antipsychotic-induced extrapyramidal side effects, has suggested involvement of the muscarinic neurotransmitter system in cognitive symptoms of psychosis (Everitt and Robbins, 1997). Administration of these anti-muscarinic agents in healthy volunteers similarly induces pronounced cognitive impairments (Vingerhoets et al., 2017). Moreover, regular treatment of Parkinson's disease with muscarinic receptor antagonists frequently induces cognitive deficits as an unwanted side effect (Xiang et al., 2012). These effects are to be predominantly mediated by the muscarinic M_1_ receptor subtype due to its high expression in critical regions for cognition (i.e., dorsolateral prefrontal cortex (DLPFC), hippocampus and striatum) (Cortes et al., 1987).

Evidence for lower M_1_ receptor expression in psychotic disorders comes from post-mortem studies showing reduced expression rates of the M_1_ receptor subtype in the DLPFC. No evidence for reductions in other important regions for cognition, such as the hippocampus and striatum have been found (Dean et al., 2000; Scarr et al., 2007). Importantly, a recently developed M_1/4_ receptor preferring agonist xanomeline showed improvements in cognition, most prominently in verbal learning and short term memory, in subjects with schizophrenia. Additionally, this drug improved both positive and negative symptoms suggesting an upstream involvement of M_1__/__4_ receptor functioning in psychotic disorders (Miller et al., 2016; Shekhar et al., 2008).

In-vivo SPECT studies have shown support for lower M_1_ receptor binding in psychotic disorders compared to control subjects (Lavalaye et al., 2001; Raedler, 2007). The study by Lavalaye et al. used ^123^I-iododexetimide (^123^I-IDEX) as a radiotracer (Lavalaye et al., 2001). Although ^123^I-IDEX has a high binding affinity for both M_1_ and M_4_ receptor subtypes, studies in muscarinic receptor knock-out mice have shown a significant reduction in ^123^I-IDEX binding in the frontal cortices (including DLPFC) in M_1_ receptor knock-out mice, but not in M_4_ receptor knock-out mice, validating that in-vivo ^123^I-IDEX binding in the DLPFC will predominantly reflect binding to the M_1_ receptor subtype (Bakker et al., 2015). Neither of the two SPECT studies (Lavalaye et al., 2001; Raedler, 2007) however, examined the relationship between lower M_1_ binding in the DLPFC and level of cognitive impairment in their psychotic patients. Because postmortem studies have singularly shown reduced M_1_ receptor expression in the DLPFC in psychotic disorders, the current study sought to examine M_1_ receptor binding in the DLPFC in relation to cognitive functioning in psychotic disorders using ^123^I-IDEX SPECT. Additionally, exploratory assessments were done to investigate the relative contribution of ^123^I-IDEX binding to M_1/4_ in the hippocampus and striatum, to cognition. As administration of xanomeline predominantly showed improvements in verbal learning and memory, we hypothesized that subjects with lower muscarinic M_1_ binding in the DLPFC would be related to greater impairments in these domains.

## 2 Methods

### 2.1 Participants

We included 30 medication-free subjects diagnosed with a psychotic disorder. Subjects were recruited from early detection programs for psychosis and through newspaper advertisements. Ethical approval was obtained from the Medical Ethical Committee of the Academic Medical Center in Amsterdam. Approval was obtained to scan subjects with a psychotic disorder, but was not granted for control subjects. The study is registered in the Dutch clinical trial registry under ID: NTR5094. Informed consent was obtained from all participants after study procedures and risks were explained. All assessments were done on the same day.

Participants were included if they met the criteria for a psychotic disorder according to the Comprehensive assessment of symptoms and history (CASH) interview (Andreasen et al., 2000), were between 18 and 40 years old, and antipsychotic medication free. Subjects being treated with low dosages of antipsychotic medication underwent a wash-out period (5 times the mean terminal elimination half-life of the specific antipsychotic) prior to participation. Only subjects using non-cholinergic antipsychotics were eligible for washout. Subjects with a bipolar disorder or psychotic depression were excluded. Additional exclusion criteria were: onset of psychotic disorder could be no >12 years prior to scanning, allergy to iodine tablets, contraindications for magnetic resonance imaging (MRI), recreational drug-use in the past 4 weeks, use of anticholinergic medication, and pregnancy in females. Participants had to abstain from alcohol and nicotine 24 h before scanning. Urine tests were utilized to test for drug intoxication and pregnancy.

### 2.2 Clinical variables assessed

A shortened version of the Wechsler Adult Intelligence Scale (WAIS) was administered to all participants to estimate level of intellectual functioning (Wechsler, 2008). Psychotic symptom severity at time of scanning was assessed using the positive and negative symptom scale (PANSS) (Kay et al., 1987), and level of social functioning and depressive symptoms using the Social Functioning Scale (SFS) (Birchwood et al., 1990) and Beck Depression Inventory (BDI-II), respectively (Beck et al., 1961). Nicotine use was assessed using the composite international diagnostic interview (CIDI). All assessments were done by trained clinical psychologists.

### 2.3 Cognitive assessment

Cognitive functioning was assessed using the Cambridge Neuropsychological Test Automated Battery (CANTAB) validated for psychotic disorders (Haring et al., 2014). The battery assesses eight cognitive domains delineated by the Measurement And Treatment Research to Improve Cognition in Schizophrenia (MATRICS) to be most prominently affected in psychotic disorders (Rodríguez-Jiménez et al., 2012). These domains are (1) visual learning and memory, (2) verbal learning and memory, (3) working memory, (4) vigilance and attention, (5) processing speed, (6) set shifting (7), reasoning and problem solving, (8) and social cognition. Cognitive assessment was performed on the same day as scanning. For an overview see Table 1.

**Table 1:** Overview of CANTAB subtests assessed.

Subtest	Cognitive domain
Paired Associate Learning (PAL)	Visual learning and memory of figure -place associations
Verbal Recognition Memory (VRM)	Verbal learning and memory
Spatial Working Memory (SWM)	Working memory
Rapid Visual Processing (RVP)	Attention and vigilance
Reaction Time (RTI)	Processing speed
One Touch Stockings of Cambridge (OTS)	Problem solving and reasoning
Emotion Recognition Test (ERT)	Social cognition

### 2.4 ^123^I-IDEX SPECT-imaging

Quantification of M_1_ receptor binding in the DLPFC was done using SPECT with the radiopharmaceutical ^123^I-IDEX. Additionally, ^123^I-IDEX was used to assess binding in the hippocampus, caudate nucleus, and putamen. Radio-synthesis of ^123^I-IDEX has been described extensively elsewhere (Bakker et al., 2015; Lavalaye et al., 2001). Each patient was pretreated with potassium iodide to block thyroid uptake of free radioactive iodide, and then received a bolus injection of approximately 185 MBq (5 mCi ^123^I-IDEX; specific activity >95%, radiochemical purity >95%). Subjects were scanned 6 h post injection, as specific binding in humans in the frontal cortex then reaches a plateau reflecting a pseudo-equilibrium condition (Boundy et al., 1995).

Static ^123^I-IDEX SPECT imaging was performed on a brain-dedicated tomographic SPECT camera (inSPira HD Neurologica, Boston, USA), with the following parameters: acquisition time per slice 180 s; slice thickness of 4 mm, slices were acquired from the level of the cerebellum up to the vertex (total acquisition approximately 60 min). An adult head computed tomography (CT) template was manually aligned in a rigid transformation and used for attenuation correction. An iterative expectation maximization algorithm tailored to the unique method of sampling across the field-of-view with a point spread function correction was used to reconstruct the data into 3D images. Spatial smoothing was accomplished using a 3 mm filter.

M_1_ receptor binding in the DLPFC was quantified as binding potential (BP_ND_) (Innis et al., 2007). The M_1_ BP_ND_ was calculated as the ratio of specific binding (Bs) in the DLPFC to nonspecific binding (Bn) binding as follows: BP_ND_ = (total binding in ROI – Bn)/Bn (Innis et al., 2007). Additionally, M_1/4_ binding (also defined as M_1_ BP_ND_) was assessed in M_1/4_-rich hippocampus, caudate nucleus and putamen gray matter, important regions for cognition. Non-specific binding (nonspecific binding + free radioligand) was measured in the cerebellar gray matter, which is devoid of M_1_ receptors (Muller-gartner et al., 1992).

For high resolution, anatomical localization of each region of interest (ROI) a structural T1 weighted MRI image (MPRAGE: voxel size 1.0 × 1.0 × 1.0 mm^3^, sagittal orientation, FOV = 256 × 240, TR = 7.0, TE = 3.2, 180 slices) was acquired for each patient on a Philips Ingenia 3.0 Tesla system (Phillips, Best, The Netherlands).

### 2.5 Image analysis

**Fig. 1:**
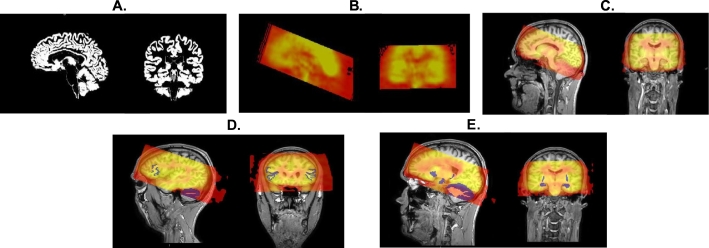
Panel A. shows gray matter segmentation image derived from the T1 structural MRI image used for co-registration of the ^123^I-IDEX SPECT scan. Panel B. ^123^I-IDEX SPECT scan of the same subject showing high cortical binding with no binding in white matter and cerebrospinal fluid. Panel C. ^123^I-IDEX SPECT scan co-registered to subject's own T1 structural MRI image; darker areas are over white matter tracts, ventricles, and cerebellum showing no ^123^I-IDEX binding. Panel D. In blue masks created from a segmented FreeSurfer image of the dorsolateral prefrontal cortex (DLPFC) (region of interest B_s_), used for the assessment of M_1_ binding, and cerebellar gray matter (B_n_), overlaid over the SPECT scan in pseudo colors. Panel E. In blue, masks used for the assessment of M_1_ binding (B_s_) in the hippocampus, caudate nucleus and putamen.

Co-registration of SPECT images to structural T1 images was performed according to the method described by Abi-Dargham and co-workers (Abi-Dargham et al., 2002) using the Statistical Parametric Mapping 12 (SPM12) software (Wellcome Trust Center for Neuro-imaging, London, UK), implemented in Matlab (Mathworks, Sherborn, MA, USA). In short, the T1 weighted image was segmented into white matter, gray matter, and cerebrospinal fluid images. Due to the fact that the cortical gray matter has the highest expression of M_1_ receptors, and consequently of ^123^I-IDEX binding, the gray matter segmentation image was binarised to create a mask to which the ^123^I-IDEX SPECT image was co-registered (Fig. 1).

Masks for the DLPFC, hippocampus, caudate nucleus, putamen and cerebellar gray matter were created using FreeSurfer version 5.3 software. Details are extensively described elsewhere (Fischl, 2012). FreeSurfer uses a surface based image processing pipeline to reconstruct the brain's cortical surface from structural MRI data, allowing for subject-specific gray matter segmentation important for concise determination of specific and non-specific binding (Klauschen et al., 2009). Regions were identified according to two morphological components (surface area + thickness = volume) using the Desikan and Killiany, and Destrieux atlases. Identified ROIs were then binarised to create a mask (Desikan et al., 2006; Fischl et al., 2002). Masks were multiplied against the co-registered SPECT image and mean counts of ^123^I-IDEX per mask were measured. The DLPFC mask included gray matter of the segmentation of the inferior frontal gyrus (angular part), dorsal part of the superior frontal gyrus, and the middle frontal gyrus. Fig. 1 depicts image analysis used to determine specific and non-specific ^123^I-IDEX binding.

### 2.6 Statistical analyses

Statistical analyses were conducted using SPSS release 20 for Windows (SPSS Inc. Chicago, IL, USA). Normality of distribution of dependent variables was verified using Kolmogorov-Smirnov and Shapiro-Wilk test. Robust regression and outlier removal (ROUT) method was used for outlier detection with an average false discovery rate of <1% (Motulsky and Brown, 2006). Sample gender distribution was tested against population prevalence using a non-parametric chi-square test. A median split analysis and independent samples *t*-test was used to assess performance on the CANTAB.

A correlation analysis was used to assess relationship between age, age of onset of psychotic disorder, duration of illness, nicotine use, and M_1_ BP_ND_ in the DLPFC, hippocampus, and striatum. Associations between categorical variables gender, subtype of psychotic disorder, item scores on the CASH, SFS, and BDI and M_1_ BP_ND_ were tested using a Spearman rank correlation.

*Z*-score based composite scores were computed for each CANTAB subtest to compute a performance measure for each cognitive domain and total overall cognitive performance on the CANTAB. A bivariate correlation analysis was used to test association between IQ and overall cognitive performance on the CANTAB and each cognitive domain. Relationship between regional M_1_ BP_ND_ and overall performance on the CANTAB and cognitive domain (see Table 1) was also assessed using a correlation analysis. Findings were corrected for multiple comparisons across the cognitive domains using a Holm-Bonferroni correction. Reported *p*-values are corrected for multiple comparisons. Additionally, a correlation analysis was used relationship between regional M_1_ BP_ND_ and psychotic symptom severity. Pearson's correlation coefficient r, and goodness of fit R^2^ are reported.

## 3 Results

### 3.1 Demographics

Majority of subjects (67%) included had only suffered a first psychotic episode and had an illness duration below 5 years (Table 2). 36% reported a period of nicotine use over the last year. 5 subjects were antipsychotic naïve, and 3 underwent a washout (*n* = 3; haloperidol (1 mg), quetiapine (200 mg) and flupentixol (0.5 mg)). The sample included more male subjects than female subjects although this difference did not statistically differ from gender prevalence of psychotic disorders in the general population (*p* = 0.70) (Jackson et al., 2013). No significant differences were found on any clinical indices between the male and female subjects. Patients had an average IQ of 100, and reported minimal symptoms of depression and social dysfunction. At time of scanning, the severity of psychotic symptoms were mild. Subjects showed moderate scores on global assessment of functioning, and relatively high scores in social functioning for subjects with a psychotic disorder. Summary of sample demographics and clinical composition are displayed in Table 2.

**Table 2:** Sample demographics and clinical composition.

	N	
Total included	30	

Gender (male/female)	20/10	

	Mean	SD

Age (yrs)	28.47	5.39
Age of onset (yrs)	20.90	6.49
Duration of illness (months)	56.07	42.25
Duration unmedication till scan (months)	38.40	41.16
IQ	99.97	15.14
Nicotine use (cigarettes per day)^⁎^	6.20	10.40
Psychotic symptoms at time of scanning		
PANSS positive scores	12.13	4.9
PANSS negative scores	12.00	5.14
PANSS general psychiatry	23.67	6.60
Other		
BDI-II total (max 63; 0–13 minimal symptoms)	13.13	11.07
SFS total (max 135; 76–86 range)	86.91	7.22
GAF general score	53.73	16.46
Psychotic disorder subtype	N	
Schizophrenia	12	
Schizophreniform Disorder	2	
Schizo-affective	3	
Psychosis NOS	13	
Number of psychotic episodes: 1/2/3/4	20/6/3/1	
% of subjects in early phase (0–5 years illness duration)	67%	

IQ: estimated intelligence quotient * at peak use in the last 12 months PANSS: Positive and Negative Syndrome Scale BDI: Beck Depression Inventory SFS: Social Functioning Scale GAF: general assessment of functioning SD: standard deviation NOS: not otherwise specified.

No significant association was found between gender, age, age of onset of psychotic disorder, duration of illness, or subtype of psychotic disorder and M_1_ BP_ND_ in DLPFC, hippocampus and striatum. In addition, no significant relationship was found between amount of cigarettes smoked per day and M_1_ BP_ND_ in these regions.

### 3.2 M_1_ receptor BP_ND_ and cognitive performance

**Fig. 2:**
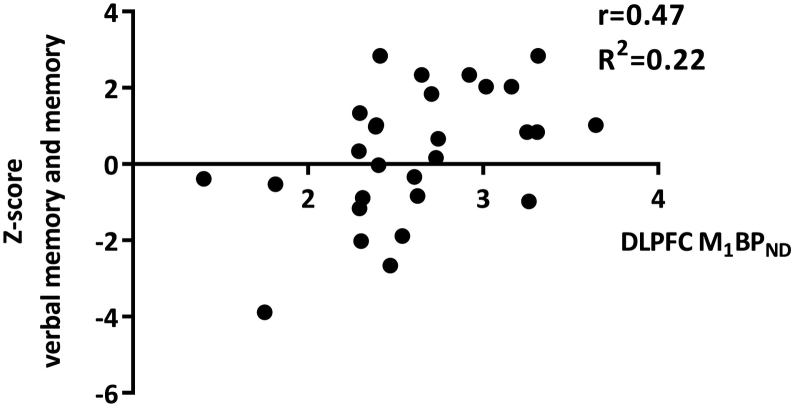
Lower M_1_ receptor binding potential (BP_ND_) in the dorsolateral prefrontal cortex (DLPFC) was related to lower verbal learning and memory capacity. r = Pearson's correlation coefficient; R^2^ = goodness of fit.

**Fig. 3:**
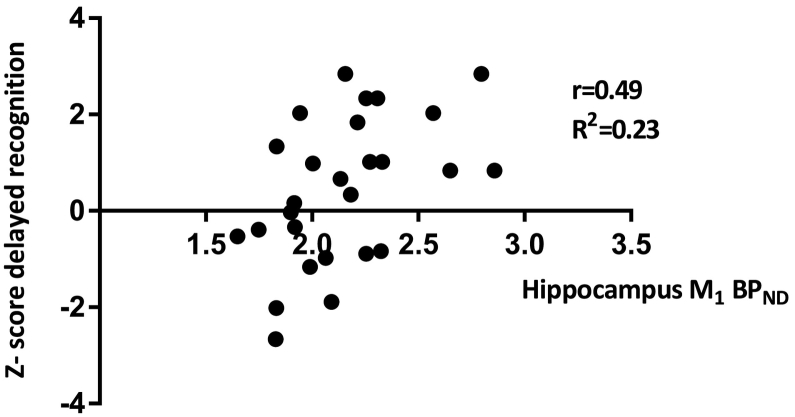
Decreased hippocampal M_1_ BP_ND_ was associated with worse delayed recognition of verbal information. r = Pearson's correlation coefficient, R^2^ = goodness of fit.

Subjects scored 1 standard deviation lower than a normative control group in overall cognition, and median split analysis showed significant difference between low and high overall cognition scores (*t* = −7.6 (29), *p* < 0.001) (Cambridge Cognition, 2006). Estimated IQ significantly predicted overall cognition scores on the CANTAB (*p* = 0.0001, *r* = 0.67), and on the cognitive domains executive functioning (*p* = 0.039, *r* = 0.38), working memory (*p* = 0.032, *r* = 0.40), and attention (*p* < 0.001, *r* = 0.62). A partial correlation analysis was conducted for these domains. Muscarinic M_1_ BP_ND_ in the DLPFC did not significantly predict overall cognitive performance on the CANTAB, but did predict verbal learning and memory domain, scores. Lower M_1_ BP_ND_ was related to significantly worse verbal learning and memory scores when correcting for multiple comparisons across cognitive domains (*p* = 0.01, *r* = 0.47; R^2^ = 0.22 (Fig. 2)). Analysis further demonstrated a significant association between lower hippocampal M_1_ BP_ND_ and worse delayed recognition of learned verbal stimuli when corrected for multiple comparisons (*p* = 0.001, *r* = 0.49, R^2^ = 0.23, (Fig. 3)). M_1_ BP_ND_ in the caudate nucleus and putamen showed no significant relationship with any of the cognitive domains measured by the CANTAB.

### 3.3 M_1_ receptor BP_ND_ and clinical variables

**Fig. 4:**
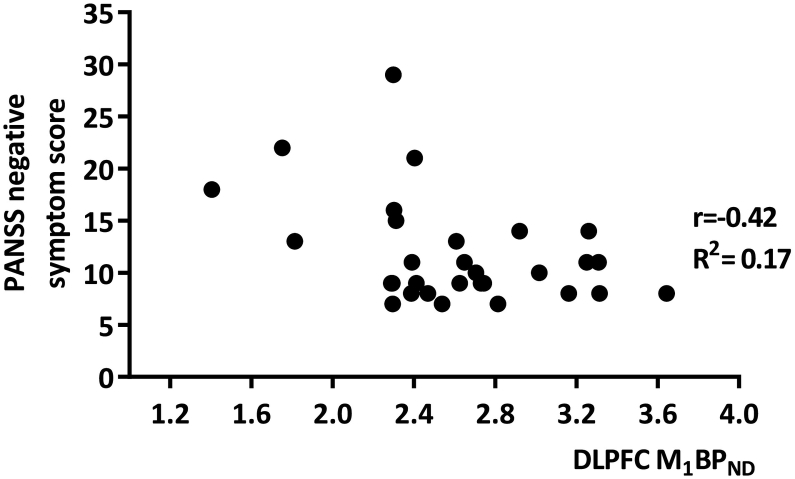
Low M_1_ BP_ND_ in the DLPFC was significantly related to increased severity and presence of negative symptoms measured by the positive and negative syndrome scale (PANSS) at time of scanning. No significant correlation was found between M_1_ BP_ND_ and positive symptoms or general psychiatry at time of scanning. r = Pearson's correlation coefficient R^2^ = goodness of fit.

Assessment of psychotic symptom severity showed lower M_1_ receptor BP_ND_ in the DLPFC was significantly associated with greater negative symptom severity (*p* = 0.01, *r* = −0.42, R^2^ = 0.17 (Fig. 4)). Similar significant association was found between lower hippocampal M_1_ BP_ND_ and negative psychotic symptom severity (*p* = 0.036, *r* = −0.38 R^2^ = 0.15). No significant association was found between M_1_ BP_ND_ in the DLPFC and hippocampus and positive symptom severity or general psychiatry scores, nor with reported depressed symptoms. Results did show lower M_1_ BP_ND_ in the hippocampus was significantly correlated to lower scores on the independent competence subscale of the SFS (*p* = 0.048, *r* = 0.36). No significant correlation was found with other factors of social functioning. In addition, a significant negative correlation between M_1_ BP_ND_ in the caudate nucleus and motor symptoms (*p* = 0.017, *r* = −0.43), and a trend significant negative correlation between M_1_ BP_ND_ in the putamen and motor symptoms (*p* = 0.059, *r* = −0.349) as rated by the CASH. These findings did not survive correction for multiple comparisons.

### 3.4 Post-hoc mediation analysis

As results show that M1 BPND is related to both verbal learning and memory deficits and negative symptoms severity post-hoc mediation analysis was conducted to further explore the relationship between these two variables, and whether M_1_ BP_ND_ has a mediating influence. Mediation analysis was done using PROCESS implemented in SPSS (Hayes, 2012). Results showed significant inversed relationship between verbal learning and memory scores and negative symptom severity (*t* = −2.39, *p* = 0.024), but no support for a mediation effect by M_1_ BP_ND_ in the DLPFC (*t* = −1.7, *p* = 0.10, BootLLCI = −0.18, BootULCI = 0.024, PM = 0.1). Theoretically impairments in verbal learning and memory could also play a mediating role in presence of increased negative symptom severity in subjects with lower M_1_ BPND. This was also tested through a mediation analysis. Again, a significant inverse relationship between M1 BPND and negative symptom severity (*t* = −2.88, *p* = 0.0076) was found but verbal learning and memory capacity was not a significant mediating factor (*t* = −1.01, *p* = 0.33, BootLLCI = −0.13, BootULCI = 0.035, PM = 0.6).

## 4 Discussion

The current study is the first to investigate how muscarinic M_1_ receptor binding in the DLPFC is related to cognitive functioning and clinical symptoms in medication-free subjects with psychosis. Results showed a significant positive correlation between muscarinic M_1_ BP_ND_ in the DLPFC and verbal learning and memory performance and a negative correlation with negative symptom severity.

In line with expectation, present results showed a significant relationship between lower M_1_ receptor binding in the DLPFC and worse verbal learning and memory performance (Fig. 2). The term BP refers to the ratio of *B*_*max*_ (here muscarinic M_1_ receptor density in the DLPFC) to *K*_D_ (here affinity of the radiotracer for muscarinic M_1_ receptors) (Innis et al., 2007). If we assume that this affinity is constant, changes in BP_ND_ reflects changes in muscarinic M_1_ receptor density. Consequently, lower M_1_ density in the DLPFC may be associated with worse verbal learning and memory performance. These findings are compatible with improvements in verbal learning and memory found under M_1/4_ agonism by xanomeline in patients with schizophrenia, and deficits in verbal learning and memory found under antagonism by biperiden, which has a 10 fold higher affinity for the M_1_ receptor over the other subtypes, in both healthy controls and psychotic disorders (Shekhar et al., 2008; Veselinović et al., 2015). Although ^123^I-IDEX in in-vitro studies also shows a relatively high affinity for the M_4_ subtype, expression rates of M_4_ in the DLPFC of humans are low and preclinical studies showed no changes in ^123^I-IDEX binding in the frontal cortex of M_4_ knock-out mice, suggesting preferential binding of ^123^I-IDEX to M_1_ receptors in this brain area (Bakker et al., 2015). The data thus supports that the efficacy of xanomeline to improve verbal learning and memory in psychotic disorders may be more attributable to its M_1_ agonist properties, rather than M_4_. Further extending on this notion, the M_1_ specific positive allosteric modulator GSK1034702 has shown to improve verbal learning and (immediate) memory in an acute nicotine abstinence model of cognitive dysfunction in human subjects (Nathan et al., 2013).

A less anticipated finding was the association between lower M_1_ binding in the DLPFC and increased negative symptom severity (Fig. 4). Although underlying mechanisms of negative symptoms are poorly studied, one study demonstrated increased negative symptom severity under increasing muscarinic receptor occupancy by antipsychotic olanzapine measured with the M_1/4_ SPECT tracer ^123^I-iodoquinuclidinyl-benzilate (^123^I-IQBN) in patients with schizophrenia (Raedler et al., 2000). Olanzapine is an antagonist at all muscarinic receptor subtypes, with the highest binding affinity in-vitro for the M_1_, M_4_, and M_5_ subtype (Bymaster et al., 1996). Due to the M_1_ subtype being the most abundant expressed receptor in the forebrain, it seems plausible that this effect was largely driven by occupancy at the M_1_ receptor (Levey et al., 1991). Our results support this postulate. Similarly, improvements in negative symptoms by xanomeline thus may also be attributable to its M_1_ agonist properties (Shekhar et al., 2008).

Cognitive and negative symptoms in psychotic disorders have been found interrelated, and data to date supports the hypothesis that these symptoms have separable but related etiologies, although more conclusive studies are still needed (Harvey et al., 2006). In line with these previous findings post hoc mediation analysis showed a significant inverse effect between lower verbal learning and memory capacity and increased negative symptom severity, but M_1_ BP_ND_ does not seem to mediate this relationship, suggesting that it is involved differently in the etiology of these symptoms.

Aside from significantly reduced M_1_ receptor expression rates found in the DLPFC, a post-mortem study also identified a subgroup of patients (25%) within their sample with a more marked reduction of M_1_ receptors (75%) in the DLPFC (Scarr et al., 2009). These results suggest that this subgroup may represent a separate endophenotype of schizophrenia presenting clinically with pronounced cognitive deficits. Results from our first in-vivo measures of M_1_ BP_ND_ in the DLPFC found no support for such a subgroup, finding no bimodal distribution of M_1_ BP_ND_ in the DLPFC in subjects with the schizophrenia psychotic disorder subtype. That being said certain considerations should be addressed. Although the current study included 12 subjects with a schizophrenia diagnosis this may have been too few to determine a subgroup. Moreover, the majority of subjects (67%) were in an early phase of the disorder, potentially this subgroup with marked loss of M_1_ receptor expression appears later in the disease etiology or is associated with chronic episodes. Lastly, the post-mortem measures may have been confounded by a life time use of antipsychotic treatment. Future assessments should be done to evaluate this more conclusively.

In an exploratory analysis of hippocampal and striatal M_1_ BP_ND_, the present results showed an association between lower hippocampal M_1_ BP_ND_ and worse verbal learning and memory performance, with a significant association with worse delayed verbal recognition. In contrast to the DLPFC, the expression of M_1_ and M_4_ receptors are both high in the hippocampus and striatum (Levey et al., 1991). Consequently, the M_1_ BP_ND_ in the hippocampus and striatum may be a combination of both M_1_ and M_4_ receptor binding. So, the association between M_1_ BP_ND_ in the hippocampus and memory consolidation and retrieval may be driven by the M_1_ or M_4_ receptor, or both. Interestingly, both muscarinic M_1_ and M_4_ receptors may play a role, through indirect modulatory processes, on glutamate and dopamine, both linked to learning and memory consolidation (Dudai et al., 2015; Hasselmo, 2006). Lower M_1_ expression is linked to loss of long term potentiation through reduced potentiation of glutamatergic *N*-methyl-d-aspartate (NMDA) receptors in the hippocampus and may be suboptimal in subjects with lower muscarinic M_1_ BP_ND_ (Collingridge et al., 2013). This result is fitting with the beneficiary effects of switching from olanzapine (possessing M_1_ receptor antagonistic properties) to non-M_1_ antagonistic antipsychotics on verbal learning and memory (Weiner et al., 2004). Interestingly, a SPECT study found lower NMDA receptor binding in the hippocampus in patients with schizophrenia compared to matched healthy controls (Newcomer et al., 1999). In addition, administration of the NMDA antagonist ketamine to healthy volunteers shows a dose-dependent negative effect on verbal memory performance (Collingridge et al., 2013). Thus, lower performance on the verbal learning and memory task in the lower binding M_1_ binding subjects could also, in part be explained by lower NMDA receptor expression or a combination of both. Whether lower NMDA receptor expression in the hippocampus in psychotic disorders is due to loss of afferent M_1_ receptor signaling is unclear. In future studies, it may be of interest to assess both M_1_ and NMDA receptors in patients suffering from a psychotic disorder, to test the relative contribution of lower muscarinic functioning and NMDA neurotransmission to deficits in verbal learning and memory.

Secondly, the current study also found exploratory results with regard to lower muscarinic receptor binding in the striatum and increased presence of motor symptoms. These findings did not survive correction for multiple comparisons, and are in need of further investigation in which motor symptoms are better objectified. These results do, however, give lead to the discussion on the origin of the highly prevalent motor symptoms in first episode psychosis patients (van Harten et al., 2015). Psychotic disorders have been associated with a striatal presynaptic hyperdopaminergic state and many patients respond well to post-synaptic dopamine D_2_ receptor blockade by antipsychotics, however it is an oversimplification to explain the whole clinical presentation of psychosis by this mechanism. In fact, this finding is particularly related to the positive symptoms of schizophrenia, and to patients that respond well to antipsychotics. In treatment refractory patients there seems to be no indication of increased striatal dopamine synthesis (Kim et al., 2016). Similarly, it is unclear whether striatal presynaptic hyperdopaminergic state also occurs in motor areas of the striatum, or whether this is more related to associative regions of the striatum. Results highlight the need for more extensive investigation into regional specific alterations in psychotic disorders, particularly because one ^18^F-DOPA PET study has measured considerably lower presynaptic dopamine function in a catatonic patient with schizophrenia (Hietala et al., 1995). These results beckon that subjects with motor symptoms may tend towards a hypodopaminergic state in motor regions of the striatum. Dopaminergic and cholinergic systems are highly interconnected, in that dopamine release inhibits acetylcholine release. Thus, if it turns out that patients with psychosis and motor symptoms are indeed in a more hypodopaminergic state, acetylcholine release may be increased. Consequently, a higher release of acetylcholine may cause a lower binding of the radiotracer ^123^I-IDEX to M_1_ receptors, and as such may explain our finding of a negative relationship between motor signs and M_1_ receptor binding.

### 4.1 Strengths and limitations

One of the major strengths of the current study was that it is the first study to assess in-vivo M_1_ binding in relation to cognition. This was done using a validated and M_1_ preferring SPECT tracer and delineation of the DLPFC was done using subject's own anatomical MRI images giving high anatomical accuracy compared to conventional manual methods. In addition, we assessed a relatively large group of subjects with psychotic disorders that were medication-free.

Absence of permission to scan healthy control subjects made it impossible to evaluate whether reported M_1_ binding in the DLPFC was significantly reduced in the presently studied subjects. However, there is already some evidence for reduced muscarinic receptor binding in psychosis. A single small study using ^123^I-IDEX SPECT in subjects with schizophrenia being treated with risperidone (no affinity for muscarinic receptors) showed significantly reduced M_1_ BP_ND_ in the frontal cortex (mean BP_ND_: 2.9) compared to healthy controls, which are also highly comparable to binding potentials reported in the current study in the DLPFC (mean BP_ND_: 2.7) (Lavalaye et al., 2001). Moreover, lower muscarinic receptor availability in psychosis has been found using the non-selective muscarinic SPECT tracer ^123^I-IQNB (Raedler et al., 2003). Although comparisons of patient groups with healthy control subjects are of scientific interest, this strategy does not deal with the heterogeneity within patient populations. For this reason, we investigated the broader spectrum of psychotic disorders rather than solely schizophrenia. The way forward may not be identifying markers for psychosis as a diagnosis but markers to stratify groups within psychosis. Such a strategy will ultimately help develop more personalized treatment options that may even cross nosological boundaries (Kapur et al., 2012; Scarr et al., 2015). As such, findings from the current study corroborate that the muscarinic M_1_ receptor may be an important biomarker for cognitive and negative symptoms in psychotic disorders which are typically difficult to manage in clinical practice.

In the current study, we measured BP_ND_ and consequently, we cannot discriminate whether a lower M_1_ binding reflects lowered M_1_ receptor expression or a higher synaptic acetylcholine in the DLPFC. Future studies are needed to evaluate whether ^123^I-IDEX binding is sensitive to change in synaptic acetylcholine levels. Post-mortem studies found indications that subjects with lower M_1_ receptor expression respond less well to both orthosteric and allosteric agonist stimulation, therefore future neuroimaging studies are paramount to assess in-vivo M_1_ receptor status and responsiveness to new M_1_ receptor targeting drugs (Dean et al., 2016; Salah-Uddin et al., 2009). Lastly, it is relevant for future studies to further examine the involvement of striatal muscarinic M_1_ receptor neurotransmission in relation to a more extensive objectification and quantification of motor symptoms in psychotic disorders. This may help identify mechanisms involved in the presentation of these symptoms and may show relevance as proxy to predict therapeutic response to antipsychotics.

## 5 Conclusion

The current study showed that lower cortical M_1_ receptor expression in the DLPFC plays a role in learning and memory performance and negative symptoms in psychotic disorders, and importantly shows that this link is already present in early stages of the disorder and in subjects with mild psychotic symptoms. Additionally, although post mortem findings report unaltered M_1_ receptor expression rates in the hippocampus and striatum, current in-vivo findings in our study suggest lower M_1_ receptor functioning in these regions may play a role in negative symptoms, cognition and presence of motor symptoms. Findings warrant additional investigation of M_1_ mediated effects underlying these symptoms particularly with drugs like xanomeline and M_1_ positive allosteric modulators being developed for the treatment of psychosis.
